# Effects of dietary garlic oil and black seed oil supplementation on growth performance, immunity, and immune-related gene expression in broiler chickens

**DOI:** 10.3389/fvets.2026.1864649

**Published:** 2026-07-15

**Authors:** Mohammad D. Obeidat, Ghassan A. Khafajah, David G. Riley

**Affiliations:** 1 Department of Animal Production, Faculty of Agriculture, Jordan University of Science and Technology (JUST), Irbid, Jordan; 2 Department of Animal Science, Texas A&M University, College Station, TX, United States

**Keywords:** essential oils, gene expression, growth performance, immunity, blood profile

## Abstract

Phytogenic feed additives encompass a broad category of plant-derived compounds that are used to improve animal health and production performance. Essential oils, as a volatile subclass of phytogenics extracted from plant materials, are recognized for their antimicrobial, antioxidant, and immunomodulatory properties and have been increasingly investigated as alternatives to antibiotic growth promoters in broiler nutrition. The study objective was to evaluate the effect of garlic and black seed oils on live broiler growth performance, immunity, and related gene expression. A total of 560 day-old Indian River broiler chicks were assigned randomly in a completely randomized design to one of four dietary groups, with each treatment consisting of 7 replicates (20 birds per replicate): control (CON; *n* = 140), garlic oil supplemented diet (200 mg/kg feed garlic oil, GO; *n* = 140), black seed oil supplemented diet (150 mg/kg feed black seed oil, BSO; *n* = 140), and the mixed oils group (200 mg/kg, 150 mg/kg for garlic and black seed oils, respectively, GO+BSO; *n* = 140). Supplementation with GO or BSO individually enhanced final body weight (*p* = 0.0356) and feed conversion ratio (*p* = 0.0407). White blood cell counts were lower in the treatment groups (*p* = 0.035). Serum triglycerides, VLDL, and AST levels were significantly reduced in the treatment groups (*P* < 0.05). *Clostridium perfringens* counts were lower in the treatment groups (*p* = 0.0006). Gene expression analysis revealed significant downregulation of pro-inflammatory cytokines (IL-6, IL-8, and TNF-α) and AvBD9, alongside upregulation of LEAP-2 and SLC11A1 in liver and spleen tissues. Dietary supplementation with garlic oil or black seed oil significantly enhanced broiler growth performance, lipid metabolism, immune function, and gut microbiota balance. The combined treatment (GO+BSO) did not produce additive or synergistic effects, suggesting overlapping mechanisms of action. These findings support GO and BSO as effective multifunctional alternatives to antibiotic growth promoters in sustainable poultry production.

## Introduction

Poultry production is among the fastest-expanding sectors of the animal production industry, essential to global food security and nutritional standards (1). Because of the increased concerns over antibiotic residues on human health, the poultry industry in different countries has banned the use of antibiotic growth promoters (2). This encouraged the researchers to seek alternatives of plant origin. Phytogenic feed additives, particularly essential oils, have emerged as viable alternatives, with demonstrated capacity to improve growth performance, nutritional digestibility, and available energy in broiler diets (3), enhance immunological function, boost digestive enzyme secretion (4), and reduce intestinal pathogen colonization through gut microbiota modulation (5). Garlic and black seed oils are well-known essential oils with proven benefits to broilers health and performance. Among the most extensively studied phytogenic preparations, garlic oil and black seed oil have shown documented benefits to broiler health and performance.

Garlic is considered a herbal medicine and spice for the prevention and treatment of a variety of diseases (6). Recent research shows that garlic (*Allium sativum*) includes various quantities of vitamins (thiamine, riboflavin, and niacin) and enzymes (allinase, peroxidase, and myrosinase), as well as 17% protein, 0.8% fat, and 3% minerals. Additionally, it has 0.2% volatile oils, and thiosulfinate compounds (mainly allicin) which are released during the plant's powder processing (7). These components have a variety of benefits, including antibacterial activity, stimulation of animal digestive systems, antioxidants, anticoccidial activity, increased formation of digestive enzymes, and improved utilization of digestive products through improved liver functions (8).

Black seed (*Nigella sativa* L.) is an aromatic annual plant cultivated globally, especially in the Middle East and South Asia (9). It has a considerable oil content of 26–34% (10) containing most of its pharmacologically active components (11). Its protein profile is dominated by glutamic acid, arginine, and aspartic acid. The primary fatty acids are linoleic acid (polyunsaturated) and oleic acid (monounsaturated), with palmitic acid as the dominant saturated fraction (12). The principal volatile bioactives—thymoquinone and dithymoquinone—exhibit anti-tumor, anti-inflammatory, and antimicrobial properties (13).

To the best of our knowledge, this study is among the first to simultaneously evaluate the effects of garlic oil and black seed oil—individually and in combination—on broiler growth performance, lipid metabolic parameters, gut microbial populations, hematological indices, and multi-gene immune expression profiles in both hepatic and splenic tissues within a single experimental framework. The inclusion of SLC11A1 and LEAP-2 as novel immunological gene expression endpoints, alongside classical pro-inflammatory cytokine profiling (IL-6, IL-8, TNF-α, and AvBD9), represents a meaningful extension of current knowledge on phytogenic-mediated innate immune modulation in broilers. Given the fact that there are bioactive compounds in both garlic and black seed oils (8, 14), we hypothesize that the use of garlic and black seed oils in the diet of broiler chickens will improve live growth performance, immunity, and related gene expression in liver and spleen tissues.

The objectives of the current study were to evaluate the effect of garlic oil and black seed oil inclusion in broiler diets on live growth performance, immunity, and related gene expression.

## Materials and methods

### Location, birds, and experimental design

A total of 560 day-old Indian River broiler chicks were randomly assigned to one of four dietary groups. The diet was formulated to meet or exceed broiler nutrient requirements to both the National Research Council (NRC) guidelines (15), and the specific recommendations from the broiler strain's producer (16) (Table 1). The phases were starter (1–14 days) and grower (15–33 days). Diets were formulated to be isonitrogenous and isoenergetic, suitable for each phase. Birds were housed in floor pens (20 chicks each) (pen dimensions, 2 × 1.24 m) in a semi-closed house at the Agricultural Research and Training Unit at Jordan University of Science and Technology, 7 pens per diet and were fed their diets daily at 09:00 with 23 h of light and 1 h of dark per day during the whole experiment. Chicks were fed their diets *ad libitum* and had free access to fresh water throughout the experiment. Temperature was kept at 32 °C on the first day and reduced gradually using cooling fans to reach 21 °C at day 33. The relative humidity inside the house ranged from 45 to 50. Light intensity ranged from 20 to 30 lx for the first 5 days and then reduced to 10 lx until day 33. All pens were covered with new wood shavings as bedding litter.

**Table 1:** Starter and grower diet composition and nutrients analysis.

Ingredient	Starter diet 1–14 day	Grower diet 15–33 day
Ingredient %		
Corn	63.3	63.5
Soybean meal	32.04	30.825
Fish meal	0.25	0.25
Meat and bone meal	0.375	0.5
Oil	0.7	1.5
DL- Methionine	0.225	0.25
L-Lysine	0.125	0.15
Salt	0.23	0.215
Limestone	1.435	1.61
mono-calcium phosphate	0.77	0.65
Sodium carbonate	0.1	0.1
Bio-emulsifier	0.05	0.05
Phytase	0.01	0.01
Protease	0.03	0.03
Vitamin and mineral premix^1^	0.2	0.2
Anticoccidial	0.1	0.06
Antifungal	0.06	0.1
Total	100	100
Calculated nutrients		
Metabolizable energy (kcal/kg)	2,937.96	2,992.31
Crude protein	20.67	20.2
Crude fat	2.57	2.57
Crude fiber	2.73	2.69
Calcium	0.86	0.92
Available phosphate	0.4	0.37
Sodium	0.23	0.22
Dig Threonine	0.66	0.64
Dig Lysine	1.04	1.03
Dig Methionine	0.51	0.5
Dig cysteine	0.27	0.26
Dig Tryptophan	0.22	0.22
Dig Isoleucine	0.79	0.77
Dig Valine	0.84	0.82
Dig TSAA	0.57	0.56
Dig Arginine	1.19	1.15

^1^ Zinc: 120 mg, Iron: 80 mg, Manganese: 120 mg, Cobalt, 1 mg Copper: 10 mg, and Iodine, 2.5 mg, for vitamin and trace mineral premix supplied the following per kg of diet: 1,852 IU vitamin D3, 0.23 mg biotin, 11 IU vitamin E, 5,512 IU vitamin A, 0.06 mg vitamin B12, 1.87 mg menadione (K3), 0.44 mg thiamine, 1.32 mg vitamin B6, 3.75 mg riboflavin, 0.22 mg folic acid, 5.95 mg d-pantothenic acid, and 34.17 mg niacin.

A single tube feeder and an automated bell drinker were included with every pen. For all treatments, birds were raised until they reached 33 days of age under standard commercial conditions.

### Treatment design

**Figure 1:**
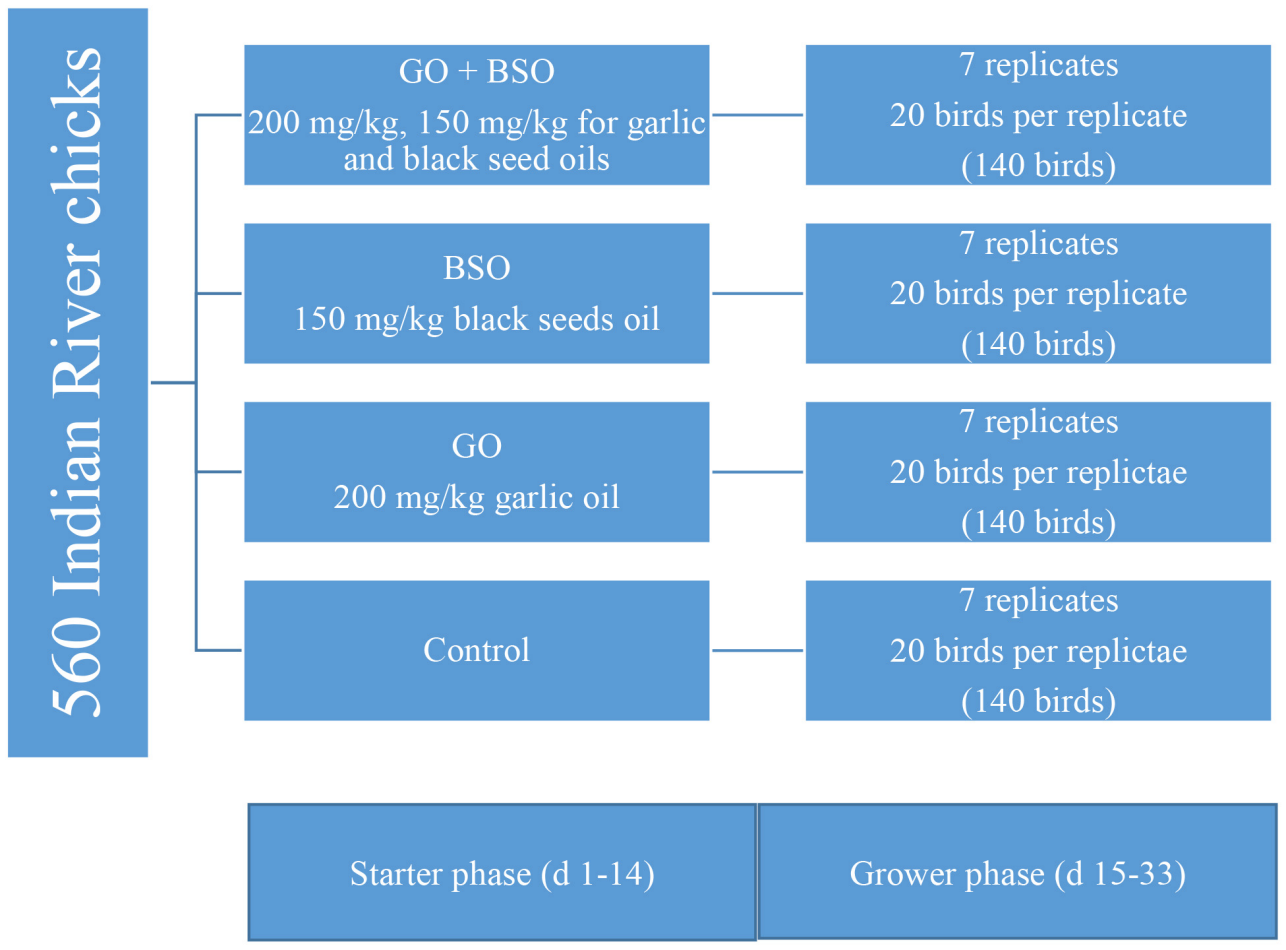
Experimental design.

Diet formulations and management practices were implemented in accordance with the Indian River management guidelines as implemented at Jordan University of Science and Technology. Four diets were applied to the birds: control (CON; *n* = 140), 200 mg/kg garlic oil (GO; *n* = 140), 150 mg/kg black seed oil (BSO; *n* = 140), and the mixed oils group (200 mg/kg, 150 mg/kg for garlic and black seed oils, respectively) (GO+BSO; *n* = 140), Figure 1. Both oils' purity and quality were validated by Certificates of Analysis (CoA) supplied by the supplier. Garlic oil (natural, food grade; Sigma-Aldrich, USA, Product No. W250320) satisfied certified specifications for refractive index (1.550–1.590), specific gravity (1.040–1.110), infrared spectrum conformance to structure, and heavy metal limitations within acceptable ranges. Black seed oil (cold pressed, crude; Batch No. 31F23, Green Fields, Jordan) was certified for relative density (0.916), refractive index (1.468), iodine value (116.40), saponification value (182.99), moisture content (0.09%), and heavy metal levels within acceptable limits, with microbiological testing also confirming the absence of *E. coli, Salmonella*, and *Staphylococcus aureus*.

The supplementation doses were selected based on published literature. Garlic oil was included at 200 mg/kg feed, within the established growth-promoting range of 150–250 mg/kg (17, 18). Black seed oil was included at 150 mg/kg feed, consistent with the optimal effective dose range for broiler performance of 100–200 mg/kg (19–22).

The initial day's temperature was maintained at 33 °C, and by day 33, it was reduced gradually to 21 °C. Body weight (BW) and feed intake (FI) were recorded weekly. The feed conversion ratio (FCR) was calculated as the ratio of total feed intake to body weight gain (BWG).

### Data collection

#### Growth performance

The experimental protocol was divided into two chronologically distinct phases: **Starter** (1–14 days) and **Grower** (15–33 days). At the beginning of each week, feed was weighed and provided to each replicate within each treatment group. At the end of each week, feed residues were collected and weighed, and weekly feed intake was calculated as the difference between the feed offered and the residual feed.

Chicks were individually weighed upon arrival (day 1) and subsequently at the end of each experimental week. Body weight gain (BWG) and feed conversion ratio (FCR) were computed on a weekly basis according to the following equations:


$$\begin{array}{c} \text{BWG }\left(\text{g}/\text{day}\right)=\left({\text{W}}_{\text{t}}\;-\;{\text{W}}_{0}\right)/\Delta \text{t}, \end{array}$$

where W_t_ = final body weight (g), W0 = initial body weight (g), and Δt = duration (days).


$$\begin{array}{c} \text{FCR }=\text{ Σ }\text{F}\text{e}\text{e}\text{d}\;\text{i}\text{n}\text{t}\text{a}\text{k}\text{e}\left(\text{g}\right)\;/\text{Σ}\;\text{B}\text{W}\text{G}\;\left(\text{g}\right). \end{array}$$

#### Tissue sampling

After slaughter on day 33, liver and spleen tissue samples weighing approximately 100 mg were collected from 7 birds per treatment (one bird per replicate). This resulted in 14 tissue samples per treatment group (7 liver + 7 spleen), for a total of 56 tissue samples across all four treatments (14 samples × 4 treatments). Quantitative real-time polymerase chain reaction (RT-qPCR) was conducted to assess the mRNA expression levels of the housekeeping gene **β-actin** using a real-time thermal cycler (CFX96 Touch™ Real-Time PCR Detection System, Bio-Rad, California, USA).

#### Primers design table

The sequences for interleukin-6 (*IL-6*), interleukin-8 (*IL-8*), tumor necrosis factor-α (*TNF-*α), solute carrier family proteins (*SLC11A1*), avian beta-defensin 9 (AvBD9; formerly GAL6), and liver-expressed antimicrobial peptide-2 (*LEAP-2*) were obtained from National Center for Biotechnology Information (NCBI) database. Based on the sequences, specific primers were designed for the target genes using NCBI Primer-BLAST tool, as detailed in Table 2.

**Table 2:** Primers used for gene expression analysis using quantitative real-time PCR.

Gene^1^	Orientation	Primer	Accession number
IL-6	F	CTTCGACGAGGAGAAATGCC	NM_204628
	R	TAGCACAGAGACTCGACGTT	
IL-8	F	CTCTGTCGCAAGGTAGGACG	NM_205498
	R	GCTGAGCCTTGGCCATAAGT	
TNF-α	F	TGGCAGCTGTGGTGCAAATA	NM_204267.2
	R	TGCAGCCTTTGCAGAGATGA	
SLC11A1	F	GCCCTGCTATGGCATCATTG	U40598
	R	ACATTGCTGGCGTCAGTTTG	
GAL6	F	CTCTTCCAGGCTGCTCCAGCTTAC	AY534894
	R	TTAGGAGCTAGGTGCCCATTTG	
LEAP-2	F	CTTCTGAGACTGAAGCGGATGAC	AY534899
	R	TCACTCGGAGGCCGTTCTAAG	

^1^ Gene are IL-6 = interleukin-6, IL-8 = interleukin-8, TNF-α = tumor necrosis factor alpha, SLC11A1 = Solute carrier family 11 member 1, GAL6= Gallinacin-6, LEAP-2 = Liver expressed antimicrobial peptide 2.

#### RNA extraction, cDNA synthesis, and qRT-PCR analysis

Following homogenization of spleen and liver tissues, total RNA was isolated from the supernatants using TRIzol-chloroform extraction followed by isopropanol precipitation. RNA pellets were resuspended in DEPC-treated RNase-free water (Ambion, Austin, TX, USA), and residual genomic DNA was eliminated using DNase I (Ambion, Austin, TX, USA). RNA purity (A260/A280 ratio) and concentration were determined spectrophotometrically (NanoDrop™).

Two micrograms of total RNA were reverse transcribed into cDNA using the iScript™ cDNA Synthesis Kit (Bio-Rad, Hercules, CA, USA). Quantitative real-time PCR was performed on a CFX96 Touch™ Real-Time PCR Detection System (Bio-Rad) using iQ™ SYBR^®^ Green Supermix (Bio-Rad).

Each 20 μL reaction contained:

- 10 μL SYBR Green master mix
- 2 μL each of each primer (forward and reverse) (10 pmol/μL)
- 2 μL cDNA template
- 4 μL nuclease-free water

Thermal cycling conditions were as follow:

1. Initial denaturation and activation: 50 °C (2 min), 95 °C (15 min)
2. Amplification (40 cycles): 95 °C (10 s), 55 °C (30 s), 72 °C (10 s)
3. Melting curve analysis: starting at 95 °C (20 s)

Primer efficiencies were determined using a 5-point serial dilution standard curve (1:2 dilutions) and confirmed in the range of 90–110% (R^2^ > 0.98) for all primer pairs. Melt curve analysis was performed following each run to confirm amplicon specificity and the absence of primer dimers. Relative gene expression was calculated using the 2−ΔΔCt method (23) normalized to β-actin as the reference gene. β-Actin stability was confirmed using geNorm analysis across all treatment groups (M-value < 0.5).

TNF-α expression was assessed in splenic tissue only, based on its established predominant expression in macrophage-rich lymphoid tissues in avian species (24, 25). The liver was prioritized for downstream antimicrobial peptide and nutrient transporter gene expression (LEAP-2, AvBD9, SLC11A1), which are biologically more relevant hepatic endpoints.

### Analysis of blood parameters

On day 33 of the experiment, blood samples were collected from 7 randomly selected birds per treatment for hematological and serum biochemical analysis. Approximately 4 mL of blood was collected from the jugular vein of each bird. Sample intended for hematology were collected using a sterile syringe into clean sample bottles containing an anticoagulant, while the remaining 2 mL of blood for serum biochemical analysis was collected into a plain sterile bottle. Samples were transferred to the JUST research laboratory using an appropriate ice box.

Hematological parameters were analyzed using an H360 fully automated three-part hematology analyzer (operating temperature: 15 to 30 °C; open-vial reagent stability: 2 to 8 °C). Serum biochemical indices were determined with a SMT-120 chemistry analyzer, calibrated to a resolution of 0.001 absorbance units (Abs) and an absorbance range of 0 to 3 Abs. Analytical conditions were maintained at a relative humidity of 30 to 70%, with a sample volume requirement of 90 to 120 μL, incubation temperature of 37 °C, and an assay run time of approximately 12 min per sample.

### Bacterial count

One bird per replicate (n = 7 per treatment) was slaughtered on day 33. A section of intestine from the distal duodenum to the ileocecal junction was removed; ~1 g of digesta was collected, homogenized in 0.9% sterile saline (1:10 w/v), and subjected to serial 10-fold dilutions (10^−1^ to 10^−6^). For TBC, 1 mL aliquots were plated onto Plate Count Agar (Oxoid CM0325) at 37 °C for 24–48 h aerobically. For *Clostridium perfringens*, appropriate dilutions were plated onto TSC agar (Oxoid CM587) under anaerobic conditions (AnaeroGen sachet, Oxoid) at 37 °C for 48 h. Black sulfite-reducing colonies were subcultured onto 5% sheep blood agar to confirm double-zone hemolysis; identity was confirmed by Gram staining (Gram-positive rods) and absence of motility. Results were expressed as log10 CFU/g digesta (26).

## Statistical analysis

Statistical analysis was performed using the General Linear Model (GLM) procedure in SAS 9.4 (SAS Inst., Inc., Cary, NC). The model included treatment as a fixed effect to evaluate growth performance and the relative mRNA expression levels of *IL-6, IL-8, TNF-*α, *SLC11A1*, AvBD9 (formerly *GAL-6*), and *LEAP-2*. Statistical significance was set at *P* < 0.05, and mean differences were evaluated using the Tukey-Kramer multiple comparison test.

## Results

### Growth performance

The effects of dietary supplementation with garlic oil (GO), black seed oil (BSO), and their combination (GO+BSO) on broiler growth performance are summarized in Table 3. Evaluated parameters included body weight (BW), feed intake (FI), body weight gain (BWG), and feed conversion ratio (FCR) across growth phases: Starter (d 1–14), Grower (d 15–33).

**Table 3:** Growth performance of broilers as affected by Garlic and Black seed oils supplementation.

TRT^1^						
ITEM^2^	Control	Garlic	Black seed	Mixed	SEM^3^	*p*-value
BW (g)						
*1*	46.000	45.667	45.833	45.667	0.443	*0.9411*
*14*	460.301 ^a^	466.063 ^a^	433.577 ^b^	429.341 ^b^	8.317	*0.0097*
*33*	2,181.667 ^ab^	2,306.667 ^a^	2310 ^a^	2,156.667 ^b^	43.508	*0.0356*
d 1–14						
*FI (g)*	565.703	573.871	570.009	545.121	8.945	*0.1390*
*BWG (g)*	414.301 ^a^	420.396 ^a^	387.744 ^b^	383.674 ^b^	8.234	*0.0091*
*FCR*	1.368 ^b^	1.368 ^b^	1.471 ^a^	1.421 ^ab^	0.0254	*0.0263*
d 14–33						
*FI (g)*	2,202.304	2,181.695	2,219.685	2,117.965	37.566	*0.2723*
*BWG (g)*	1,721.366 ^c^	1,840.604 ^ab^	1,876.423 ^a^	1,727.326 ^bc^	39.677	*0.0230*
*FCR*	1.282 ^a^	1.189 ^b^	1.184 ^b^	1.226 ^ab^	0.0247	*0.0407*
d 1–33						
*FI (g)*	2,768.007	2,755.566	2,789.695	2,663.083	41.390	*0.1763*
*BWG (g)*	2,135.667 ^bc^	2,261.000 ^ab^	2,264.167 ^a^	2,111.000 ^c^	43.486	*0.0354*
*FCR*	1.298	1.221	1.233	1.262	0.0203	*0.0648*

^1^ Values are LSMeans ± SEM; *n* = 7 replicates, TRT: garlic oil was included in the diet at a rate of 200 mg/kg. Black seed oil was included in the diet at a rate of 150 mg/kg. Mixed diet was supplemented with garlic and black seed oils 200 mg/kg, 150 mg/kg, respectively.

^2^ FI = feed intake, BWG = body weight gain, FCR = feed conversion ratio.

^3^ SEM = standard error of the mean.

^a, b, c^ Mean values within rows with different superscripts differ significantly (*p* < 0 .05).

No significant differences in initial BW (d 1) were observed among groups (*P* = 0.941). During the starter phase (d 1–14), BW was significantly higher in the GO (466.06 ± 8.317 g) and control groups (460.30 ± 8.317 g) compared to the BSO (433.58 ± 8.317 g) and GO+BSO (429.34 ± 8.317 g). However, feed intake showed a numerical reduction in birds fed GO+BSO compared to those fed GO, although this difference was not statistically significant. Body weight gain was significantly higher in the control (414.3 ± 8.234 g) and GO (420.4 ± 8.234 g) groups than in the BSO (387.7 ± 8.234 g) and GO+BSO (383.7 ± 8.234 g) groups. Feed conversion ratio was lower (*P* = 0.0263) in the control (1.368) and GO (1.368) groups compared to BSO (1.471), whereas the GO+BSO group was intermediate (1.421).

During the grower phase (d 15–33), feed intake was comparable among all groups (P = 0.2723). However, body weight gain was significantly greater in birds fed BSO (1876.4 ± 39.677 g) and GO (1840.6 ± 39.677 g) than in the control (1721.4 ± 39.677 g) and the GO+BSO (1727.3 ± 39.677 g). Feed conversion ratio was significantly lower in the GO (1.189) and BSO (1.184) groups compared to the control (1.282), whereas the GO+BSO group was intermediate (1.226).

Over the entire experimental period (d 1–33), final BW at day 33 was significantly higher in the GO (2306.67 ± 43.508 g) and BSO (2310.00 ± 43.508 g) groups compared to the GO+BSO (2156.67 ± 43.508 g), whereas the control group was intermediate (2181.67 ± 43.508 g). Cumulative feed intake was not significantly affected by dietary treatments. Body weight gain was significantly higher in birds supplemented with BSO (2264.2 ± 43.486 g) and GO (2261.0 ± 43.486 g) compared with the control (2135.7 ± 43.486 g) and the GO+BSO (2111.0 ± 43.486 g). Although overall feed conversion ratio differences did not reach statistical significance (P = 0.0648), birds fed GO (1.221) and BSO (1.233) exhibited numerically superior feed efficiency compared with those in the control (1.298) and the GO+BSO (1.262) groups.

### Blood biochemical indicators

#### Blood biochemical parameters

Dietary supplementation with garlic oil (GO), black seed oil (BSO), or their mixture (GO+BSO) significantly influenced several blood biochemical indices in broilers (Table 4).

**Table 4:** Blood parameters of broilers as affected by Garlic and Black seed oils supplementation.

TRT^**1**^						
**ITEM** ^2^	**Control**	**Garlic**	**Black seed**	**Mixed**	**SEM** ^3^	***p-value***
Urea (mmol/L)	0.763 ^a^	0.218 ^b^	0.615 ^a^	0.440 ^ab^	0.111	*0.0162*
Cholesterol (mg/dL)	184.83	168.67	174.33	168.40	6.283	*0.2598*
Triglycerides (mg/dL)	201.73 ^a^	123.67 ^b^	103.65 ^b^	93.28 ^b^	16.852	*0.0011*
HDL (mg/dL)	79.33 ^b^	78.33 ^b^	86.33 ^a^	88.20 ^a^	2.356	*0.0206*
LDL (mg/dL)	65.15	65.55	67.23	62.10	3.350	*0.7802*
VLDL (mg/dL)	40.35 ^a^	24.73 ^b^	20.73 ^b^	18.66 ^b^	3.370	*0.0011*
ALT (U/L)	3.50	3.17	1.83	2.80	0.635	*0.3077*
AST (U/L)	992.67 ^a^	686.83 ^b^	656.83 ^b^	764.60 ^ab^	79.423	*0.0310*
Protein (g/dL)	3.25	3.17	3.18	3.14	0.117	*0.9277*
Albumin (g/dL)	1.50	1.48	1.48	1.48	0.052	*0.9929*
Globulins (g/dL)	1.75	1.68	1.70	1.66	0.077	*0.8734*

^1^ Values are LSMeans ± SEM; *n* = 7 replicates, TRT: garlic oil was included in the diet at a rate of 200 mg/kg. Black seed oil was included in the diet at a rate of 150 mg/kg. Mixed diet was supplemented with garlic and black seed oils 200 mg/kg, 150 mg/kg, respectively.

^2^ HDL= High-density lipoprotein, LDL = low-density lipoprotein, VLDL = very-low-density lipoprotein, ALT = alanine aminotransferase, AST = Aspartate aminotransferase.

^3^ SEM = standard error of the mean.

^a, b^ Mean values within rows with different superscripts differ significantly (*p* < 0.05).

Broilers fed GO exhibited reduced serum urea concentrations compared to the control group (P = 0.0162), whereas the GO+BSO and the BSO groups were intermediate. Triglyceride (TG) concentrations were markedly lower (*P* = 0.0011) in birds receiving GO+BSO (93.28 mg/dL), BSO (103.65 mg/dL) and the GO group (123.67 mg/dL) compared with the control group (201.73 mg/dL). Consistently, Very-Low-Density Lipoprotein (VLDL) levels were significantly reduced in the GO (24.73 mg/dL), BSO (20.73 mg/dL), and GO+BSO (18.66 mg/dL) groups compared to the control group (40.35 mg/dL).

High-density lipoprotein (HDL) concentrations were significantly elevated in birds supplemented with GO+BSO (88.20 mg/dL) and BSO (86.33 mg/dL) compared with those fed GO (78.33 mg/dL) or the control diet (79.33 mg/dL). Aspartate aminotransferase (AST) activity was significantly lower in the GO (686.83 U/L) and BSO (656.83 U/L) groups than in the control group (992.67 U/L), while the GO+BSO group was intermediate (764.60 U/L). In contrast, no significant treatment effects were observed on total cholesterol (*P* = 0.2598), low-density lipoprotein (LDL; *P* = 0.7802), alanine aminotransferase (ALT; *P* = 0.3077), total protein (*P* = 0.9277), albumin (*P* = 0.9929), or globulin concentrations (*P* = 0.8734).

#### Hematological parameters

The effects of dietary supplementation with garlic oil (GO), black seed oil (BSO), and their combination (GO+BSO) on blood hematological parameters are summarized in Table 5.

**Table 5:** Hematological parameters of broilers as affected by Garlic and Black seed oils supplementation.

**TRT** ^**1**^						
**ITEM** ^2^	**Control**	**Garlic**	**Black seed**	**Mixed**	**SEM** ^3^	***p-value***
Hemoglobin (g/dL)	7.383	7.200	7.367	7.400	0.134	*0.702*
Hematocrit (%)	32.150	31.750	32.533	33.833	0.626	*0.135*
RBC ( × 106/μL)	2.403	2.335	2.432	2.403	0.062	*0.726*
WBC ( × 10^3^/μL)	4.483 ^a^	3.983 ^ab^	3.667 ^ab^	3.250 ^b^	0.279	*0.035*
Lymphocytes (%)	64.417	67.133	60.400	65.967	6.012	*0.868*
Heterophils (%)	26.196	26.617	30.358	30.450	5.538	*0.912*
H/L ratio	0.422	0.430	0.558	0.598	0.148	*0.781*

^1^ Values are LSMeans ± SEM; *n* = 7 replicates, TRT: garlic oil was included in the diet at a rate of 200 mg/kg. Black seed oil was included in the diet at a rate of 150 mg/kg. Mixed diet was supplemented with garlic and black seed oils 200 mg/kg, 150 mg/kg, respectively.

^2^ RBC = Red Blood Cell, WBC = White Blood Cell, H\L = Heterophil-to-Lymphocyte (H/L) Ratio.

^3^ SEM = standard error of the mean.

^a, b^ Mean values within rows with different superscripts differ significantly (*p* < 0.05).

Dietary oil supplementation had no significant effect on hemoglobin concentration. Mean Hb levels across treatment groups were comparable, ranging from 7.200 g/dL (GO) to 7.400 g/dL (the GO+BSO group), with no statistically significant differences observed among all treatments.

Hematocrit levels showed a tendency toward treatment-related differences with a numerical elevation in the GO+BSO group. The GO+BSO group exhibited the highest mean HCT (33.833 ± 0.626%), followed by BSO (32.533± 0.626%), control (32.150 ± 0.626%), and GO (31.750 ± 0.626%).

No significant differences were detected in RBC counts. Mean values ranged from 2.335 ± 0.062 × 106/μL (GO) to 2.432 ± 0.062 × 106/μL (BSO), with all groups remaining statistically equivalent.

Dietary treatments exerted a significant influence on WBC counts. The control group displayed the highest WBC counts (4.483 ± 0.279 × 10^3^/μL), followed by GO (3.983 ± 0.279 × 10^3^/μL), BSO (3.667 ± 0.279 × 10^3^/μL), and the GO+BSO group (3.250 ± 0.279 × 10^3^/μL). *Post hoc* comparisons indicated that WBC counts in the control group were significantly higher than those in the GO+BSO group, whereas no other differences reached statistical significance.

Lymphocyte percentages remained unaffected by dietary treatments. Mean values ranged from 60.400 ± 6.012% (BSO) to 67.133 ± 6.012% (GO), with no significant intergroup differences.

Similarly, heterophil percentages did not differ significantly among treatments. The GO+BSO group exhibited the highest value (30.450 ± 5.538%), while the control group showed the lowest (26.196 ± 5.538%).

The H/L ratio was not significantly influenced by dietary interventions. Mean ratios ranged from 0.422 ± 0.148 (control) to 0.598± 0.148 (GO+BSO group), with no statistical differences observed across all treatments.

#### Bacterial count

The impact of dietary supplementation with phytogenic feed additives (garlic oil, black seed oil, and their combination) on total bacterial count (*TBC*) and *Clostridium perfringens* counts in broilers is presented in Table 6.

**Table 6:** Influence of Phytogenic feed additives (garlic and black seed oils) on broiler bacterial load.

**TRT** ^**1**^						
**ITEM** *^2^*	**Control**	**Garlic**	**Black seed**	**Mixed**	**SEM** ^3^	***p-value***
***TBC*** **(CFU/g)**	5.66	4.78	4.81	5.99	0.35	*0.0525*
***Clostridium perfringens*** **(CFU/g)**	3.47 ^a^	1.41 ^b^	1.01 ^b^	0.41 ^b^	0.45	*0.0006*

^1^ Values are LSMeans ± SEM; *n* = 7 replicates, TRT: garlic oil was included in the diet at a rate of 200 mg/kg. Black seed oil was included in the diet at a rate of 150 mg/kg. Mixed diet was supplemented with garlic and black seed oils 200 mg/kg, 150 mg/kg, respectively.

^2^ TBC = total bacterial count.

^3^ SEM = standard error of the mean.

^a, b^ Mean values within rows with different superscripts differ significantly (p < 0.05).

The dietary inclusion of garlic oil and black seed oil in the diet resulted in numerically lower total bacterial counts compared to the control group (*P* = 0.0525).

*Clostridium perfringens* counts were significantly affected by dietary treatment. All treatment groups showed lower (*P* = 0.0006) *Clostridium perfringens* colonization levels compared with the control, with the mixed oil treatment (0.41 log10 CFU/g) showing the greatest reduction, followed by the black seed oil (1.01 log10 CFU/g) and garlic oil (1.41 log10 CFU/g).

#### Gene expression

Dietary supplementation with phytogenic feed additives (garlic oil, black seed oil, or their mixture) significantly affected the relative expression of immune-related genes in the liver and spleen of broiler chickens.

#### Liver tissue

**Figure 2:**
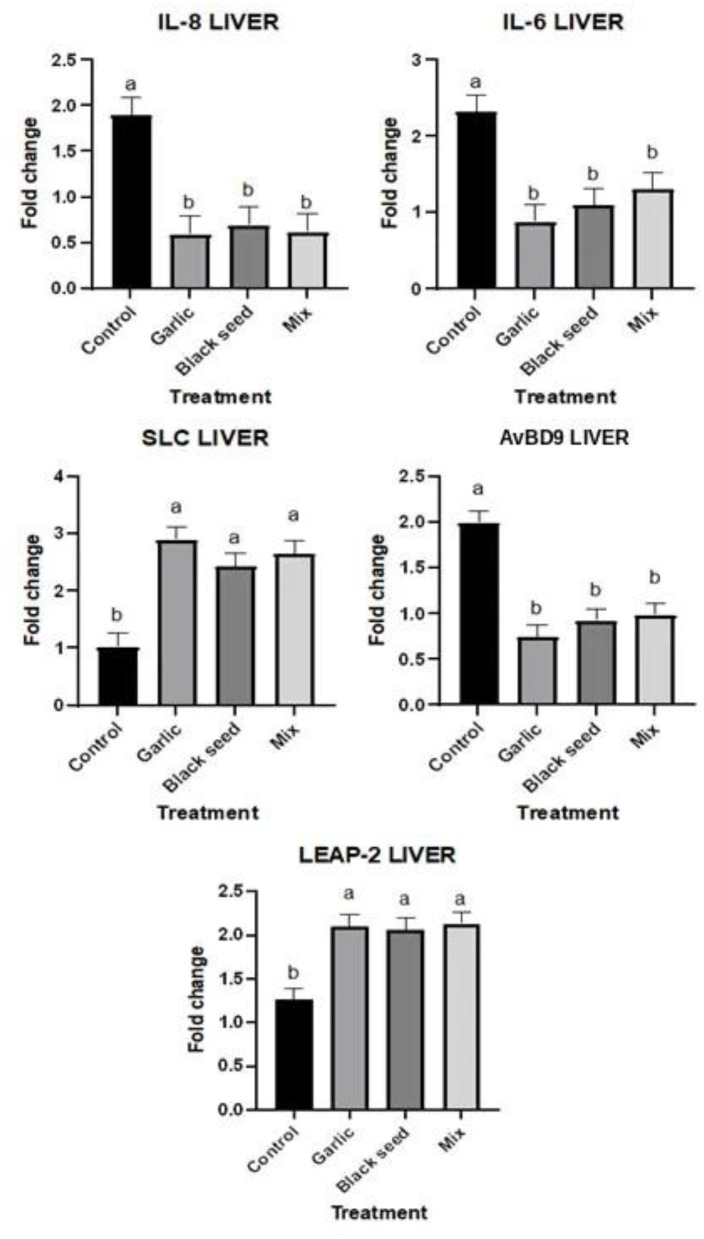
Relative mRNA expression of *IL*-*8, IL*-*6, AvBD9, SLC11A1*, and *LEAP-2* in the liver of broilers as affected by Phytogenic feed additives (garlic and black seed oils). Gene are IL-6= interleukin-6, IL-8 = interleukin-8, SLC = solute carrier, GAL6 = Gallinacin-6 (AvBD9), LEAP-2 = Liver expressed antimicrobial peptide 2.

As shown in Figure 2, ***IL-8*** expression was highest (*P* = 0.0003) in the control group (1.90 ± 0.20) compared with broiler supplemented with BSO (0.69 ± 0.20), GO (0.59 ± 0.20), or GO+BSO (0.62 ± 0.20); no significant differences were detected among the treatment groups. Similarly, ***IL-6*** expression was higher (*P* = 0.0009) in the control group (2.32 ± 0.22) compared to the BSO (1.10 ± 0.22), GO (0.88 ± 0.22), and GO+BSO (1.30 ± 0.22) groups. The expression of ***AvBD9*** (formerly ***GAL-6*** () was significantly higher (*P* < 0.0001) in the control group (2.00 ± 0.13) compared with all other treatments.

Conversely, **SLC11A1** expression was higher (*P* < 0.0001) in birds fed GO (2.89 ± 0.22), GO+BSO (2.64 ± 0.24), and BSO (2.43 ± 0.22) compared with the control (1.02 ± 0.24). Similarly, **LEAP*-2*** expression was greater (*P* = 0.0004) in the BSO (2.06 ± 0.14), GO (2.10 ± 0.14), and GO+BSO (2.13 ± 0.14) groups than in the control (1.26 ± 0.14).

#### Spleen tissue

**Figure 3:**
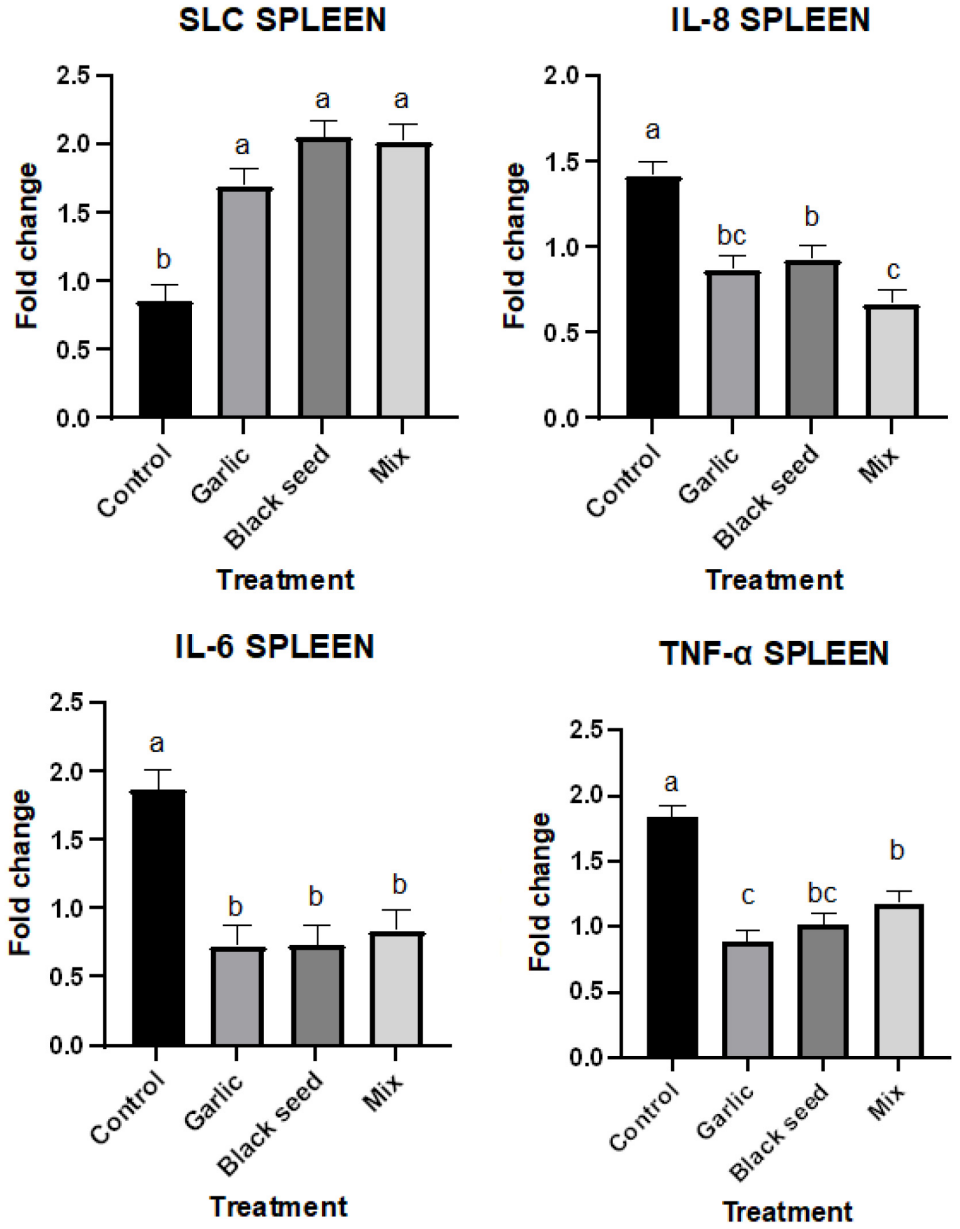
Relative mRNA expression of *TNF-*α, *SLC11A1, IL-8*, and *IL-6* in the spleen of broilers as affected by phytogenic feed additives (garlic and black seed oils). Gene are IL-6 = interleukin-6, IL-8 = interleukin-8, TNF-α = tumor necrosis factor alpha, SLC = solute carrier.

In the spleen tissue (Figure 3), **TNF*-*α** *e*xpression was the highest (*P* < 0.0001) in the control group (1.84 ± 0.09), followed by GO+BSO (1.18 ± 0.09) and BSO (1.01 ± 0.09), with GO showing the lowest expression (0.89 ± 0.09). The ***SLC11A1*** expression was significantly higher (*P* < 0.0001) in the BSO (2.05 ± 0.13), GO (1.70 ± 0.13), and GO+BSO (2.02 ± 0.13) groups compared with the control (0.86 ± 0.13).

The ***IL-8*** expression was highest (*P* < 0.0001) in the control group (1.42 ± 0.08), intermediate in BSO (0.93 ± 0.08) and GO (0.87 ± 0.08), and lowest in the GO+BSO group (0.67 ± 0.08). Finally, ***IL-6*** expression was the highest (*P* < 0.0001) in the control group (1.87 ± 0.15) compared to all phytogenic treatments.

## Discussion

This study assessed the effects of dietary supplementation with garlic oil (GO), black seed oil (BSO), and their combination (GO+BSO) on broiler growth performance, metabolic parameters, gut microbiota, and the expression of immune-related genes. The findings indicated significant improvements in growth and health parameters with individual phytogenic additives, whereas the combined supplementation did not yield synergistic effects.

The bioactive components of the garlic and black seed oils utilized in this study—primarily allicin and its derivatives for garlic oil, and thymoquinone for black seed oil—are well-documented in the literature by comprehensive GC-MS and HPLC characterization of commercially available formulations (13, 27). Functional confirmation of bioactive compound activity at the administered concentrations is provided by the steady and statistically significant improvements seen across growth, metabolic, and immune parameters in the current study, which independently supports the established compositional profiles of these oils.

### Growth performance

The enhancement of BWG and final BW observed in the GO and BSO groups, along with the improved feed conversion ratio (FCR) aligns with previous findings that document the growth-promoting properties of phytogenic feed additives (4, 28). Lee at al. (17) reported that allicin has demonstrated the ability to augment pancreatic enzyme production and exhibit antibacterial properties, resulting in enhanced feed digestibility and energy efficiency. Similarly, the thymoquinone in BSO is known for improving villus architecture and gut barrier function (29). The bioactive compounds allicin and thymoquinone have been shown to improve intestinal morphology, stimulate digestive enzyme secretion, and enhance nutrient absorption, thereby contributing to improved performance parameters (18, 30). Although the overall FCR difference across the full experimental period did not reach statistical significance (*P* = 0.0648), the consistent numerical superiority of GO (1.221) and BSO (1.233) over the control (1.298)—combined with the statistically significant FCR improvement during the grower phase specifically—suggests a biologically relevant trend that may achieve significance with larger sample sizes. These numerical differences are discussed for biological completeness and are not interpreted as statistically confirmed findings. This agrees with Saeid et al. (31) who reported that feed conversion was enhanced for birds consuming the plant premix Garlic powder and black seed in comparison to the control group. Conversely, the absence of performance improvement in the mixed GO+BSO group and the lack of synergy may occur from overlapping biological pathways or possible antagonistic interactions between the bioactive compounds, necessitating further investigation. Comparable findings were reported by Aydogan et al. (32), where 5 g/kg GO and BSO individually enhanced biochemical indices; however, their combination did not produce additional advantages.

Several mechanisms may account for the antagonistic interactions in the GO+BSO group. First, allicin and thymoquinone both potently inhibit NF-κB signaling (33, 34); their simultaneous action may have excessively suppressed this pathway, dampening downstream innate immune activation. Second, both bioactives are potent antimicrobials with overlapping targets; their combined effect may have disrupted beneficial microbiota alongside pathogens (35). Third, competitive absorption interactions between organosulfur compounds and quinones at shared intestinal transporter sites may have reduced the bioavailability of each compound. Future factorial dose-titration studies are needed to identify potential synergistic concentration thresholds.

Based on integrated analysis of all measured parameters, individual supplementation with garlic oil (GO) demonstrated the most consistently superior improvements across growth performance, hepatic enzyme profiles, and immune-related gene expression in both liver and spleen. Black seed oil (BSO) produced equivalent growth-promoting effects with complementary benefits for lipid metabolism. The combined GO+BSO treatment achieved the greatest suppression of *Clostridium perfringens* colonization but produced no additive or synergistic effects on growth or gene expression parameters. The inferior starter-phase performance of GO+BSO birds relative to GO alone may reflect the limited digestive capacity of young broilers (d 1–14). The simultaneous presence of two potent phytogenic compounds may have transiently reduced feed palatability or altered the developing gut microbiota (5) composition, resolving as adaptive tolerance developed by the grower phase.

### Blood biochemical indicators

#### Blood biochemical parameters

In chickens, very-low-density lipoprotein (VLDL) serves as the primary lipoprotein for triglyceride transport in the blood (36); however, the significant reductions in serum triglycerides (GO: 123.67, BSO: 103.65, and GO+BSO: 93.28 mg/dL) and VLDL (GO: 24.73, BSO: 20.73, and GO+BSO: 18.66 mg/dL) concentrations in the treatment groups compared to the control (201.73 and 40.35 mg/dL, respectively) indicate beneficial modulation of lipid metabolism. Thymoquinone has been reported to downregulate hepatic lipogenesis and enhance lipid clearance (37), while allicin is known to inhibit key enzymes involved in cholesterol and fatty acid biosynthesis (18). This may indicate that the antioxidant and anti-inflammatory activities in the treatment groups reduced oxidative damage in hepatocytes, thereby preventing the overproduction of triglycerides and VLDL (38). The observed increase in high-density lipoprotein (HDL) in the BSO and the GO+BSO groups (86.33 and 88.20 mg/dL, respectively) compared to the control and GO groups indicates a cardioprotective effect, potentially linked to enhanced reverse cholesterol transport. Our result agrees with Hassan (39) who reported that supplementing broiler diet with *Nigella sativa* seed significantly increased (HDL) levels. The serum triglyceride concentration in the control group (201.73 mg/dL) is within the established physiological reference range for commercial broiler chickens (100–400 mg/dL). Unlike mammals, avian species perform *de novo* lipogenesis predominantly in the liver rather than adipose tissue, and rely on VLDL-triglyceride as the primary mechanism for hepatic lipid export to rapidly growing peripheral tissues, including breast muscle. High-energy, high-carbohydrate commercial diets further promote hepatic lipogenesis and consequently maintain higher baseline circulating TG and VLDL in broilers relative to mammalian species. The significantly reduced TG in all phytogenic-supplemented groups (93–124 mg/dL) represents a beneficial modulation of hepatic lipid metabolism driven by thymoquinone-mediated downregulation of hepatic lipogenesis (37) and allicin-mediated inhibition of cholesterol/fatty acid biosynthetic enzymes (18).

Aspartate aminotransferase (AST) is an intracellular enzyme predominantly localized in hepatocytes. Hepatic damage disrupts cellular integrity, releasing AST into the systemic circulation and consequently elevating serum AST levels (40). Accordingly, the AST values recorded across all experimental groups in the present study (656–993 U/L) fall within the established physiological reference range for commercial broiler chickens (198–1,215 U/L) Tokofai et al. (41)—a range that greatly exceeds mammalian reference values owing to species-specific hepatocellular AST distribution and the inherently high metabolic activity of avian hepatocytes. Garlic oil (GO) and black seed oil (BSO) exhibit significant phytochemical activity, characterized by their antioxidant and anti-inflammatory effects (8, 26). The reduction in serum AST activity in birds supplemented with GO and BSO may reflect the hepatoprotective properties of these phytogenic agents, possibly due to their antioxidant potential and ability to stabilize hepatic cell membranes (42, 43). Although alanine aminotransferase (ALT) and total protein concentrations were unaffected, the observed biochemical trends suggest an overall improvement in liver function and metabolic homeostasis. These findings align with Elbaz et al. (44), who reported that dietary supplementation with essential oils did not significantly alter serum hepatic enzyme activities, such as ALT, or total protein levels in broilers.

The decline in serum urea levels (0.218 mmol/L) in the GO group compared to the control and the BSO groups may reflect improved nitrogen retention or reduced protein catabolism. These effects may be attributed to improved gastrointestinal health and modulation of systemic inflammation, as previously described by Elkatcha et al. (45). The lack of significant variations in serum albumin and total protein levels indicates that hepatic protein production was not influenced by either garlic or black seed oils.

The low serum urea concentration in the GO group (0.218 mmol/L) is physiologically normal in broiler chickens, in which nitrogen is predominantly excreted as uric acid rather than urea—unlike mammals. The normal avian serum urea range is 0.1–2.5 mmol/L. The significantly lower urea in GO-supplemented birds compared to control likely reflects enhanced intestinal amino acid absorption and reduced luminal proteolytic bacterial activity mediated by allicin's antimicrobial properties (8), combined with improved nitrogen retention consistent with the superior body weight gain observed in the GO group. This interpretation is supported by Elkatcha et al. (45), who reported reduced serum urea following dietary garlic extract supplementation in broilers.

#### Hematological parameters

The current findings indicate that dietary supplementation with garlic oil (GO), black seed oil (BSO), and their combination (GO+BSO) had minimal effects on most hematological parameters; however, a significant reduction in white blood cell (WBC) counts was observed in the treatment groups compared to the control.

Contrary to several studies that have reported increased leukocyte counts following garlic (18) and black seed supplementation (46), the observed reduction in leukocyte counts in the current findings suggests a potential immunomodulatory or anti-inflammatory effect rather than immunosuppression. This likely reflects a lower systemic inflammatory burden in the absence of pathogenic challenge, due to improved gut health (22, 47) or the antimicrobial properties of the supplemented oils (44, 48).

The significant reduction in total WBC in phytogenic-supplemented groups compared to control, in the absence of pathogenic challenge, is most parsimoniously interpreted as an anti-inflammatory immunomodulatory effect rather than immunosuppression (22, 47). Under standard housing conditions, the elevated baseline WBC in the control group may reflect subclinical gut inflammatory states driven by opportunistic *Clostridium perfringens* colonization—which was significantly higher in control birds (3.47 vs. 0.41–1.41 log10 CFU/g in treated groups). The simultaneous reduction in WBC, *Clostridium perfringens* counts, and pro-inflammatory cytokine gene expression (IL-6, IL-8, and TNF-α) across treated birds collectively indicates that phytogenic supplementation attenuated systemic inflammatory signaling through improved gut microbial balance (48). The physiological WBC ranges observed across all groups (3.25–4.48 × 10^3^/μL) confirm that the reductions did not represent pathological immunosuppression.

#### Bacterial count

The strong antibacterial properties of black seed and garlic oils are demonstrated by the significant decrease in *Clostridium perfringens* counts observed in all treatment groups GO+BSO (0.41 log10 CFU/g), BSO (1.01 log10 CFU/g), and GO (1.41 log10 CFU/g), which is consistent with previous reports (49, 50). The synergistic antimicrobial response found in the mixed group, with the lowest *Clostridium* levels, may result from the combined inhibitory effects of phenolic compounds (51) and quinones (52) found in garlic and black seed.

Although the reduction in the total bacterial count (TBC) did not achieve statistical significance (*P* = 0.0525), the observed numerical decline in the GO and BSO groups (4.79, and 4.81 log10 CFU/g, respectively) supports the hypothesis that phytogenic feed additives contribute to gut microbial homeostasis, potentially limiting pathogenic colonization and favoring beneficial flora (53).

#### Immune gene expression

The present study examined the immunomodulatory and metabolic effects of dietary supplementation with garlic oil (GO), black seed oil (BSO), and their combination (GO+BSO) in broiler chickens by quantifying immune-related gene expression in hepatic (IL-6, IL-8, AvBD9, SLC11A1, and LEAP-2) and splenic (TNF-α, SLC11A1, IL-6, and IL-8) tissues. GO and BSO individually downregulated pro-inflammatory cytokines (IL-6, IL-8, and TNF-α) and AvBD9 while upregulating LEAP-2 and SLC11A1; GO+BSO produced less consistent effects (Figures 2, 3).

The downregulation of IL-6, IL-8, and TNF-α across hepatic and splenic tissues indicates that these phytogenic compounds exert potent anti-inflammatory activity through inhibition of nuclear factor kappa B (NF-κB) signaling—a pathway by which allicin suppresses pro-inflammatory cytokine transcription (33), and thymoquinone attenuates pro-inflammatory mediator production (34).

Hepatic AvBD9 was significantly downregulated across all treatment groups relative to the control. Defensins belonging to the Gallinacin family are antimicrobial peptides that disrupt pathogenic microbes by binding to the negatively charged phospholipid bilayer of the cell membrane, causing bacterial cell destruction (54), and their expression is typically elevated in response to pathogenic infection (55). The GO- and BSO-induced reduction in *Clostridium perfringens* counts likely accounts for this downregulation, reflecting diminished microbial stimulation in the treatment groups—a finding consistent with Laptev et al. (56), who reported that EO supplementation significantly downregulated AvBD10, another gallinacin family member.

Hepatic LEAP-2 was significantly upregulated in GO- and BSO-supplemented birds in the absence of a pathogenic challenge, indicating proactive, diet-driven activation of innate immune defenses. The peptide exerts antimicrobial activity through membrane permeabilization of pathogenic microbes (57). Given that Casterlow et al. (58) demonstrated LEAP-2 downregulation in response to bacterial challenge in chickens, the upregulation observed here suggests that GO and BSO prime LEAP-2-mediated innate immunity through dietary bioactive constituents rather than reactive infection-driven pathways.

Dietary GO, BSO, and GO+BSO supplementation significantly upregulated SLC11A1, a membrane-bound solute carrier that facilitates cellular uptake of divalent cations and nutrients—enhancing absorptive capacity and metabolic efficiency (59), while also limiting intracellular pathogen replication within macrophages (36). The results of the current study were consistent with Wang et al. (60), who reported upregulation of SLC family members in EO-supplemented laying hens, supporting both the intestinal functional benefits and the antimicrobial potential of these phytogenic additives.

## Conclusion

Based on integrated analysis of all measured parameters, individual supplementation with garlic oil (GO) at 200 mg/kg demonstrated the most consistently superior multifunctional profile—improving broiler final body weight, grower-phase feed conversion ratio, hepatic enzyme profiles, nitrogen utilization, and immune-related gene expression in both liver and spleen tissues. Black seed oil (BSO) at 150 mg/kg produced equivalent growth-promoting effects with complementary benefits for lipid metabolism. The combined GO+BSO treatment achieved the greatest suppression of *Clostridium perfringens* colonization but produced no additive or synergistic effects on growth performance or gene expression parameters.

From a practical perspective, dietary inclusion of garlic oil at 200 mg/kg feed or cold-pressed black seed oil at 150 mg/kg feed represents a viable, antibiotic-free nutritional strategy for commercial broiler production. Both products are commercially available, fully characterized by Certificates of Analysis, and compatible with consumer demand for antibiotic-free poultry products. Further research should investigate optimal dosing strategies and efficacy under commercial rearing conditions including disease challenges and environmental stressors.

## Study limitations

Histological assessment of hepatic and splenic architecture was not performed. Future studies should include H&E histomorphometry of intestinal segments (duodenum, jejunum, colon), 16S rRNA amplicon sequencing for microbiome profiling, serum immunoglobulin quantification (IgA, IgM), multiplex cytokine ELISA, and GC-MS/HPLC characterization of specific bioactives (allicin, thymoquinone) to provide a more mechanistically complete evaluation.

## Data Availability

The raw data supporting the conclusions of this article will be made available by the authors, without undue reservation.
